# Gut Microbiome Diversity and Antimicrobial Resistance After a Single Dose of Oral Azithromycin in Children: A Randomized Placebo-Controlled Trial

**DOI:** 10.4269/ajtmh.23-0651

**Published:** 2024-01-16

**Authors:** Thuy Doan, Zijun Liu, Ali Sié, Clarisse Dah, Mamadou Bountogo, Mamadou Ouattara, Boubacar Coulibaly, Dramane Kiemde, Guillaume Zonou, Eric Nebie, Jessica Brogdon, Elodie Lebas, Armin Hinterwirth, Lina Zhong, Cindi Chen, Zhaoxia Zhou, Travis Porco, Benjamin F. Arnold, Catherine E. Oldenburg, Thomas M. Lietman

**Affiliations:** ^1^ Francis I. Proctor Foundation, University of California, San Francisco, California;; ^2^ Department of Ophthalmology, University of California, San Francisco, California;; ^3^ Centre de Recherche en Santé de Nouna, Nouna, Burkina Faso;; ^4^ Department of Epidemiology and Biostatistics, University of California, San Francisco, California;; ^5^ Institute for Global Health Sciences, University of California, San Francisco, California

## Abstract

Mass antibiotic distribution to preschool children resulted in alterations of the gut microbiome months after distribution. This individually randomized, placebo-controlled trial evaluated changes in the gut microbiome and resistome in children aged 8 days to 59 months after one dose of oral azithromycin in Burkina Faso. A total of 450 children were randomized in a 1:1 ratio to either placebo or azithromycin. Rectal samples were collected at baseline, 2 weeks, and 6 months after randomization and subjected to DNA deep sequencing. Gut microbiome diversity and normalized antimicrobial resistance determinants for different antibiotic classes were evaluated. Azithromycin decreased gut bacterial diversity (Shannon *P* < 0.0001; inverse Simpson *P* < 0.001) 2 weeks after treatment relative to placebo. Concurrently, the normalized abundance of macrolide resistance genetic determinants was 243-fold higher (95% CI: 76-fold to 776-fold, *P* < 0.0001). These alterations did not persist at 6 months, suggesting that disruptions were transient. Furthermore, we were unable to detect resistance changes in other antibiotic classes, indicating that co-resistance with a single course of azithromycin when treated at the individual level was unlikely.

## INTRODUCTION

Mass azithromycin distributions are an effective approach to eliminate trachoma and reduce childhood mortality.^1^^–^^3^ Such therapeutic benefits, however, come at the cost of selecting for antibiotic resistance.^4^^,^^5^ When mass drug administrations are discontinued, the prevalence of macrolide resistance declines over a period of months to years.^4^ Similarly, alterations in the gut microbiome can be seen with repeated distributions for up to 6 months after the last antibiotic intake.^6^ Although the effects of community antibiotic distributions last 6 months or longer, that does not imply that the effect of a single dose of azithromycin intake at the individual level would last 6 months.

Gut and Azithromycin Mechanisms in Infants and Neonates (GAMIN) is an individually randomized, placebo-controlled trial designed to evaluate changes in the gut microbiome and resistome in children aged 0–59 months after oral intake of a single dose of azithromycin. The azithromycin dosage used in this study was the same as that used in the Macrolides Oraux pour Réduire les Décès avec un Oeil sur la Résistance (MORDOR) study, in which mass drug distribution was shown to reduce childhood mortality in sub-Saharan Africa.^7^ Here, we report the short-term and longer-term impacts of azithromycin and placebo in preschool children.

## MATERIALS AND METHODS

### Trial methods.

Ethical approval for the study was obtained from the University of California, San Francisco (UCSF) Institutional Review Board and from the Comité d’Ethique pour la Recherche en Santé in Ouagadougou and the Comité Institutionnel d’Ethique at the Center de Recherche en Santé de Nouna. The study was undertaken in accordance with the Declaration of Helsinki. Written informed consent was obtained from a caregiver of each participant.

The GAMIN study took place in Nouna, the capital city of Kossi Province in Burkina Faso.^8^ It is a semi-urban city with a population of approximately 30,000 inhabitants. The study team visited households with children under the age of 5 years, based on the most recent census conducted by the Nouna Health and Demographic Surveillance Site.^9^ Caregivers were informed of the study, and interested participants were encouraged to visit the Nouna District Hospital. Children aged between 8 days and 59 months were eligible. Children were randomized in a 1:1 ratio using simple randomization to either azithromycin or placebo treatment by the trial biostatistician in R version 3.6.1 (the R Foundation for Statistical Computing, Vienna, Austria). Treatment was either a single oral dose of placebo or azithromycin (weight-based dosing for those who could not stand and height-based dosing for those who could to a target dose of 20 mg/kg). Rectal swabs were obtained at baseline (prior to treatment) and 2 weeks and 6 months after treatment. Samples were placed in the Zymo stool collection kit (Zymo Research, Irvine, CA) to preserve nucleic acid integrity, stored at −80°C in Burkina Faso, and shipped to UCSF for processing. All field and laboratory personnel were masked to the assignments.

### Laboratory methods and sequencing analysis.

For each time point, randomly chosen samples from five children were pooled according to treatment arm prior to processing. Samples were then processed for metagenomic DNA sequencing to evaluate for gut microbiome and antibiotic resistance determinants. Sample processing, sequencing, and analyses were performed as previously described.^5^^,^^6^

### Statistical methods.

Assuming 80% power and detectable effect size of 1.88 SD and no loss to follow-up, we proposed a sample size of 225 per group, totaling 450 study participants. The primary outcomes were prespecified as alpha-diversity (Shannon’s diversity, expressed as effective number) of the gut microbiome at the species level at 2 week and 6 month time points. We assumed a mean (SD) alpha Shannon’s index of 16.4 (7.12) and used a Wilcoxon rank-sum test to compare the outcomes between arms.^10^ Secondary outcomes included differential changes in the gut microbiome at the species level and the resistance determinants of macrolides and other antimicrobial classes. Differential changes in the gut microbiome at the species level and antimicrobial resistance determinants at the class level were evaluated using DESeq2 with a Benjamini-Hochberg false detection rate of < 0.01. All analyses were conducted in R, version 3.4.3. Diversity measures were calculated in the R package “vegan.”

## RESULTS

**Figure 1.**
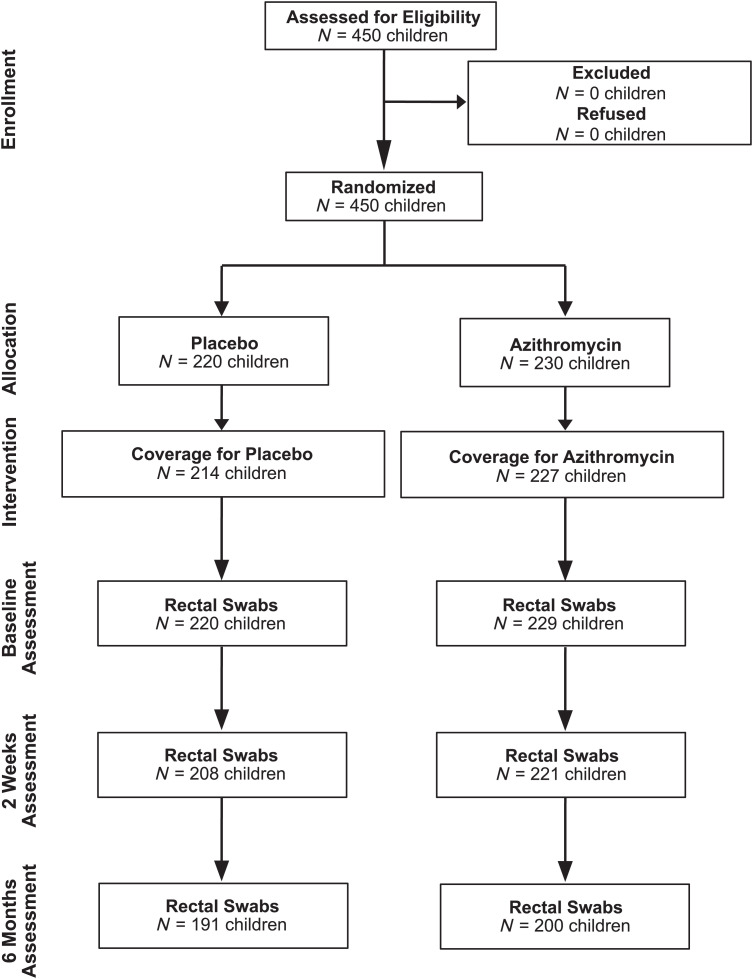
Trial profile.

In November 2019, 450 children were screened and enrolled in the study (Figure 1), with 220 randomized to receive placebo and 230 to receive a single dose of azithromycin. Treatment adherence was 97% in the placebo arm and 99% in the azithromycin arm. Six children were reported to have spit up the medication, and three children were reported to have vomited. At enrollment, the mean age was 856 days (SD ±424 days) for the placebo-treated arm and 872 days (SD ±457 days) for the azithromycin-treated arm (Table 1). Of those enrolled, a total of 449 rectal swabs were collected at baseline, 429 swabs were collected at 2 weeks, and 391 swabs were collected at 6 months. All swabs were processed and analyzed.

**Table 1:** Demographics of analyzed participants

	Placebo			Azithromycin		
Characteristic	Baseline	2 Weeks	6 Months	Baseline	2 Weeks	6 Months
Number of children	220	208	191	229	221	200
Age in days, mean (±SD)	856 (±424)	867 (±424)	866 (±411)	872 (±457)	871 (±455)	895 (±461)
Female, %	48.6	49.0	49.2	53.3	53.4	52.5

**Figure 2.**
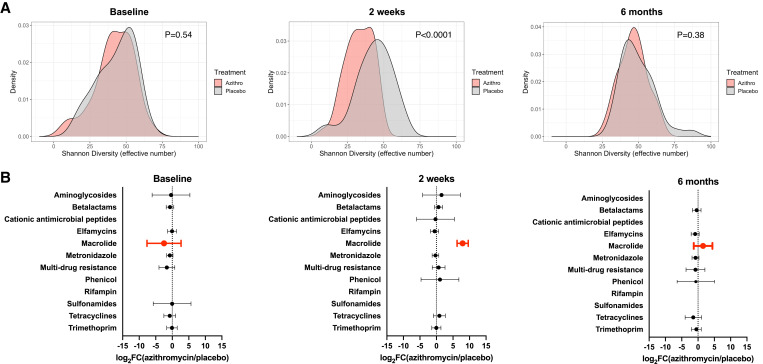
Gut microbiome diversity and antimicrobial resistance determinants of children at baseline, 2 weeks, and 6 months after a single dose of azithromycin treatment. (**A**) Density plot for inverse Shannon’s diversity index at baseline (*N* = 449), at 2 weeks (*N* = 429), and at 6 months (*N* = 391). All *P* values are permuted with 10,000 simulations and Bonferroni corrected for multiple comparisons. (**B**) Log_2_ fold change in antibiotic resistance determinants in the azithromycin-treated group compared with the placebo-treated group with associated 95% CI. Azithro = azithromycin; FC = fold-change.

Gut microbiome diversity was similar between the arms at baseline (Shannon *P =* 0.54; inverse Simpson *P =* 0.71; Figure 2A). Two weeks after treatment, the microbiome diversity was decreased in children treated with a single dose of azithromycin compared with those who were treated with placebo (Shannon *P* < 0.0001; inverse Simpson *P* < 0.001, Figure 2A). Diversity was indistinguishable by 6 months after treatment (Shannon *P =* 0.38; inverse Simpson *P =* 0.46; Figure 2A). Differential abundance analysis of the gut microbiome at 2 weeks, where there were notable perturbations, showed changes in 157 bacterial species.

The relative normalized abundance of macrolide resistance determinants in children treated with azithromycin was 243-fold higher than those in children treated with placebo (95% CI: 76-fold to 776-fold, *P* < 0.0001; Figure 2B) at 2 weeks. By 6 months, this relative increase in macrolide resistance determinants had normalized (95% CI: 1/2-fold to 21-fold, *P =* 0.67; Figure 2B). No evidence of statistically significant co-resistance to other antibiotic classes was detected (Figure 2B) either at 2 weeks or 6 months. Similarly, the diversity of the resistome was not statistically different between groups at 2 weeks (Shannon *P =* 0.25; inverse Simpson *P =* 0.20) or at 6 months (Shannon *P =* 0.85; inverse Simpson *P =* 0.62).

## DISCUSSION

We evaluated the gut microbiome and resistome of preschool children who were individually treated with antibiotics. We found that a single dose of azithromycin intake resulted in a rapid but transient decrease in microbiome diversity that was associated with a corresponding dramatic increase in macrolide resistance determinants at 2 weeks. These alterations were not detectable at 6 months after treatment.

The MORDOR trial showed that mass drug distribution of azithromycin to children younger than 5 years decreased childhood mortality in Niger, Malawi, and Mozambique.^1^ However, antibiotic resistance persisted up to 6 months after the last antibiotic distribution.^6^ Potentially more concerning was the finding of co-resistance being induced by mass drug distribution, which appeared to have saturated after eight mass administrations.^5^ In addition to macrolides, other antibiotic classes that were found to be increased included beta-lactams, aminoglycosides, metronidazole, trimethoprim, and bacitracin. Thus, individualized treatment may be desirable if the selection for antibiotic resistance is transient, as found in this study, although the efficacy of individual prophylactic treatment to reduce childhood mortality may be limited.

The gut microbiome of these children appears resilient and recovered by 6 months. In a smaller study where children in two rural communities of the Nouna District in Burkina Faso were individually randomized to receive a single course of three commonly prescribed antibiotics in the region, those receiving azithromycin showed a significant decrease in gut microbiome diversity and a relative reduction for *Campylobacter* species compared with children in the placebo arm.^11^ Here, 157 species were differentially altered between treatment arms, including several *Campylobacter* and *Clostridium* species. Although these organisms are associated with disease, in the appropriate physiological context, even commensal organisms can be pathogenic. Because these children did not take azithromycin for a gastrointestinal illness, the functional effect of the overall shift in the gut microbiome in this population is not clear, even if known pathogenic bacteria were reduced.

Other limitations of this study should be noted. First, the nearly 6-month follow-up interval after 2 weeks prevented precise characterization of the microbiome and macrolide resistance recovery kinetics. Thus, a recovery prior to 6 months would have been missed as no samples were collected between 2 weeks and 6 months after administration of azithromycin. Regardless, the relatively quick recovery of macrolide resistance to baseline was in contrast to the prolonged selection of resistance when entire communities were treated twice annually.^5^ Second, because the children were recruited from a semi-urban area of Burkina Faso, they may be healthier and more likely to be exposed to antibiotics than communities farther from the health facilities.^12^ Third, the samples of children in the same arm were randomly pooled into groups of five for processing. This testing design has several implications. Although it reduces the number of randomization units 5-fold, the pool is a weighted average of the five samples, minimizing variance.^13^ Note that the random pooling of samples from children at each time point prevented assessment of longitudinal changes within the same child over time. This question is currently being addressed by our team in another individually randomized controlled trial in which samples are collected and tested individually from children every 2 days for 2 weeks and then again at 6 months.

Here, we found no evidence of prolonged gut microbiome diversity or resistome alterations after a single dose of azithromycin intake in an individual. The effects were detectable at 2 weeks but not at 6 months. These results are reassuring, as antibiotic use remains necessary for the treatment of childhood infections worldwide.
